# Change the Qualis Criteria!^*^

**DOI:** 10.1590/S1516-31802010000400014

**Published:** 2010-07-01

Since Capes (Coordenação de Aperfeiçoamento de Pessoal de Nível Superior; Coordination Office for Improvement of University-level Personnel) published its revised Qualis criteria, the AMB (Associação Médica Brasileira; Brazilian Medical Association) has been organizing a series of meetings, held at its headquarters in São Paulo, Brazil, starting in August 2009, in partnership with the ABEC (Associação Brasileira de Editores Científicos; Brazilian Association of Scientific Editors). These meetings led to the publication of an editorial entitled "Classification of journals in the Qualis System of Capes – urgent need of changing the criteria!" The editorial was signed by 62 editors of scientific journals and published in full in all of them, and in many others, primarily within the health sciences, demonstrating that Brazil’s periodicals are more and more committed to discussing the problems that they share in common.^1^

The scientific community remains concerned about the future prospects and direction of Brazilian periodicals.^2,3^ The editors present at the meeting on March 18 were therefore in a position to evaluate the repercussions of the first editorial, which had been discussed at scientific events and meetings all over Brazil. The meeting was attended by Dr. Lilian Caló, who is scientific communication and assessment coordinator for SciELO (Scientific Electronic Library Online). She presented a comparative study of the Brazilian periodicals indexed by SciELO, according to two different criteria: the first criterion was the ISI/JCR (Institute for Scientific Information/Journal Citation Report) impact factor, which only uses journals indexed by Thomson Reuters, and the second was an index formed by simple addition of the ISI/JCR and SciELO impact factors. The SciELO impact factor, which includes all the periodicals that it indexes, significantly modifies the number of citations and, consequently, raises the Brazilian periodicals’ impact factors. Dr. Caló’s presentation illustrated this fact more clearly, showing the percentages gained by adopting the composite index. It is clear that by combining indexes, or creating equivalencies or several alternatives, improved quality ratings among Brazilian journals can be promoted, thereby raising their visibility and making international indexation more likely. It is also of concern that Brazilian researchers now prefer to publish their work in foreign journals rather than choosing domestic publications. They choose to do this because it improves the ratings of their postgraduate departments, earns them greater impact factors and increases their H index; all entirely and exclusively a result of the revised criteria adopted by Capes. Achievements in increasing the visibility and quality of Brazilian scientific production should not be assessed on the basis of articles alone, but should also focus on improving our periodicals to the point at which they are recognized internationally.

Considering that the criteria have already been set for Capes’ current triennial assessment, the assembled editors decided to draft a second editorial containing a list of suggestions for the next assessment, to be sent to the administration at Capes. The list of suggestions, which supplement those in the first editorial, is as follows:

– that the criteria used by Capes to classify periodicals be revised, adopting the composite impact factor calculated by summing the ISI/JCR and SciELO impact factors;
– that a seat on the Capes Scientific Committee be created for ABEC so that editors can be heard within the process;
– that CNPq (Conselho Nacional de Desenvolvimento Científico e Tecnológico; National Council for Scientific and Technological Development) be requested to create an "Editors’ Scholarship" program to support scientific publishing and to be awarded to the editors of journals funded by CNPq. The objective of this project would be to improve the quality of these journals by providing their editors with more time to dedicate to their editorial activities.

Additionally, the assembled editors decided to seek support for their criticisms and suggestions from the Academia Brasileira de Ciências (Brazilian Academy of Sciences), from FINEP (Financiadora de Estudos e Projetos; Funding Office for Studies and Projects) and from Federal Deputy Eleuses Vieira de Paiva. At a later date, the editors will request a detailed breakdown from CNPq of the criteria adopted for, and the results from, the distribution of the resources allocated through the Publishing Support Grants (Editais para Auxílio à Editoração). The editors intend to use these data to construct a database on the annual budgets of Brazilian periodicals, which will be useful for comparative analysis and mutual cooperation. Publication of these two editorials and promotion of discussion is one of our goals in seeking the recognition that Brazilian periodicals both need and deserve.

This editorial is signed by:

**Adagmar Andriolo**
*Jornal Brasileiro de Patologia e Medicina Laboratorial*
**Alfredo José Afonso Barbosa**
*Jornal Brasileiro de Patologia e Medicina Laboratorial*
**Arnaldo José Hernandez**
*Revista Brasileira de Medicina do Esporte*
**Aroldo F. Camargos**
*Revista Femina*
**Benedito Barraviera**
*Journal of Venomous Animals and Toxins including Tropical Diseases*
**Bogdana Victoria Kadunc**
*Surgical & Cosmetic Dermatology da Soc. Brasileira de Dermatologia*
**Bruno Caramelli**
*Revista da Associação Médica Brasileira*
**Carlos Brites**
*Brazilian Journal of Infectious Diseases*
**Dejair Caitano do Nascimento**
*Hansenologia Internationalis*
**Domingo M. Braile**
*Revista Brasileira de Cirurgia Cardiovascular*
**Dov Charles Goldenberg**
*Revista Brasileira de Cirurgia Plástica*
**Edmund Chada Baracat**
*Revista da Associação Médica Brasileira*
**Edson Marchiori**
*Revista Radiologia Brasileira*
**Eduardo de Paula Vieira**
*Revista Brasileira de Coloproctologia*
**Eros Antônio de Almeida**
*Revista da Sociedade Brasileira de Clínica Médica*
**Flávia Machado**
*Revista Brasileira de Terapia Intensiva*
**Geraldo Pereira Jotz**
*Revista Brasileira de Cirurgia Cabeça e Pescoço*
**Gianna Mastroianni Kirsztajn**
*Jornal Brasileiro de Nefrologia*
**Gilberto Camanho**
*Revista Brasileira de Ortopedia*
**Gustavo Gusso**
*Medicina Família e Comunidade*
**Ivomar Gomes Duarte**
*Revista de Administração em Saúde*
**Izelda Maria Carvalho Costa**
*Anais Brasileiros de Dermatologia*
**João Ferreira de Mello Júnior**
*Brazilian Journal of Otorhinolaryngology*
**Joel Faintuch**
*Revista Brasileira de Nutrição Clínica*
**José Antônio Baddini Martinez**
*Jornal Brasileiro de Pneumologia*
**José Antônio Livramento**
*Arquivos de Neuro-Psiquiatria*
**José Eduardo Ferreira Manso**
*Revista do Colégio Brasileiro de Cirurgiões*
**José Eulálio Cabral Filho**
*Revista Brasileira de Saúde Materno Infantil*
**José Heverardo da Costa Montal**
*Revista da Associação Brasileira de Medicina de Tráfego*
**José Luiz Gomes do Amaral**
*Revista da Associação Médica Brasileira*
**José Luiz Martins**
*Archives of Pediatric Surgery*
**Jurandyr Moreira de Andrade**
*Revista Brasileira de Ginecologia e Obstetrícia*
**Leonardo Cançado Monteiro Savassi**
*Revista Brasileira de Medicina de Família e Comunidade*
**Luís dos Ramos Machado**
*Arquivos de Neuro-Psiquiatria*
**Luiz Augusto Casulari**
*Brasilia Médica*
**Luiz Eugenio Garcez Leme**
*Geriatria & Gerontologia*
**Luiz Felipe P. Moreira**
*Arquivos Brasileiros de Cardiologia*
**Luiz Henrique Gebrim**
*Revista Brasileira de Mastologia*
**Marcelo Madeira**
*Revista Brasileira de Mastologia*
**Marcelo Riberto**
*Revista Acta Fisiátrica*
**Marcus Bastos**
*Jornal Brasileiro de Nefrologia*
**Mário Cícero Falcão**
*Revista Brasileira de Nutrição Clínica*
**Mario J. da Conceição**
*Revista da Sociedade Brasileira de Anestesiologia*
**Mauricio Rocha e Silva**
*Revista Clinics*
**Milton Artur Ruiz**
*Revista Brasileira de Hematologia e Hemoterapia*
**Milton K. Shibata**
*Arquivos Brasileiros de Neurocirurgia*
**Mittermayer Barreto Santiago**
*Revista Brasileira de Reumatologia*
**Nelson Adami Andreollo**
*Arquivos Brasileiros de Cirurgia Digestiva*
**Nivaldo Alonso**
*Brazilian Journal of Craniomaxilofacial Surgery*
**Osvaldo Malafaia**
*Arquivos Brasileiros de Cirurgia Digestiva*
**Olavo Pires de Camargo**
*Acta Ortopedica Brasileira*
**Paulo Manuel Pêgo Fernandes**
*São Paulo Medical Journal*
**Regina Helena Garcia Martins**
*Brazilian Journal of Otorhinolaryngology*
**Renato Soibelmann Procianoy**
*Jornal de Pediatria*
**Ricardo César Pinto Antunes**
*Revista da Sociedade Brasileira de Cancerologia*
**Ricardo Fuller**
*Revista Brasileira de Reumatologia*
**Ricardo Guilherme Viebig**
*Arquivos de Gastroenterologia*
**Ricardo Nitrini**
*Dementia & Neuropsychologia*
**Rogério Dedivitis**
*Revista Brasileira de Cirurgia Cabeça e Pescoço*
**Ronaldo Damião**
*Urologia Contemporânea*
**Rosângela Monteiro**
*Revista Brasileira de Cirurgia Cardiovascular*
**Sergio Lianza**
*Revista Medicina de Reabilitação*
**Sigmar de Mello Rode**
*Brazilian Oral Research*
**Tarcisio E. P. Barros Filho**
*Acta Ortopedica Brasileira*
**Wallace Chamon**
*Arquivos Brasileiros de Oftalmologia*
**Winston Bonetti Yoshida**
*Jornal Vascular Brasileiro*
**Zuher Handar**
*Revista Brasileira de Medicina do Trabalho*

## References

[1] (2010). Classificação dos periódicos no sistema Qualis da Capes - a mudança dos critérios é urgente! [Classification of journals in the Qualis system of Capes - urgent need of changing the criteria!]. Rev Assoc Med Bras.

[2] Lucena AF, Tibúrcio RV (2009). Qualis periódicos: visão do acadêmico na graduação médica [Qualis periodical: view of an academic on medical graduation]. Rev Assoc Med Bras (1992).

[3] Rocha-e-Silva Mauricio (2009). O novo Qualis, ou a tragédia anunciada: [editorial]. Clinics.

